# Concerns about Diuretics as the First-line Treatment of Hypertension in the Elderly

**DOI:** 10.14336/AD.2021.0224

**Published:** 2021-10-01

**Authors:** Gang Li, Rui Hu

**Affiliations:** ^1^Cardiology Division in Geriatric Institute, Hebei General Hospital, Shijiazhuang, Hebei, China; ^2^General Clinical Laboratory, The Second Hospital of Hebei Medical University, Shijiazhuang, Hebei, China

**To the Editor**

Based on our extensive clinical experience, we are very concerned about the choice of antihypertensive drugs in the elderly. Recently, an authoritative guide recommended the use of thiazide diuretics, calcium channel blockers (CCB), angiotensin-converting enzyme inhibitors (ACEI) and angiotensin II receptor blockers (ARB) for hypertension, and thiazide diuretics as the first-line therapy in the majority of elderly hypertensive patients. There is evidence that the use of thiazide diuretics reduces comprehensive cardiovascular outcomes in hypertensive patients over 65 years of age, and thiazide diuretics (especially chlorothiazide or indapamide) are better than other categories of drugs for older hypertensive patients [1]. Based on our lengthy clinical practice, we have learned that the basic principle of hypertension treatment is to increase urinary sodium excretion, and diuretics can counter extracellular volume expansion and sodium retention caused by hypertension [2]. However, it may likely lead to electrolyte disorders in elderly hypertensive patients [3]. Some studies have revealed that hypokalemia (up to 8%) may not only precipitate cardiac arrhythmias and related sudden death but also adynamia by muscular weakness, which increases the risk of falls. Hyponatremia (up to 17%) may contribute to fatigue, cognitive impairment, gait defects, falls, adverse effects on bone mass (e.g., osteoporosis) and fractures, which are also more common and severe in older patients [4,5]. Patients may have a higher risk of falls if they develop postural hypotension during diuretic therapy, or frequent nocturnal urination symptoms may seriously affect their rest and sleep, which lead to the lowest adherence rate (only 34.4% after 2 years) among first-line antihypertensives [6]. The fundamental goal of hypertension treatment is to control hypertension and reduce the overall risk of complications and death caused by hypertension. However, the burden of co-morbidities, quality of life, and possible contingencies after reducing blood pressure should also be considered, in addition to the basic objectives of the antihypertensive treatment.

Therefore, although we agree with the application of diuretics in the treatment of hypertension in the elderly, the concurrent diseases of elderly patients must be considered and the risk of possible adverse events after antihypertensive treatment must be carefully analyzed. Furthermore, the mode of administration of diuretics is also worth considering. For example, a study recommended to take antihypertensive drug once a day in the afternoon, which will help in controlling blood pressure, reducing water and sodium retention, and nocturia, thereby reducing the risk of adverse events [7].
